# Popliteal Aneurism

**Published:** 1876-08

**Authors:** Julius F. Miner


BUFFALO

### Medical and Surgical Journal.

VOL XVI. AUGUST, 1876. No. 1.

### Original Communications.

:o:

ART. I.—*Popliteal Aneurism. Ligature to external femoral without beneficial result. Tumor exposed, contents evacuated and vessels ligated. Cure of Aneurism and recovery without any unpleasant symptoms.* By PROF. JULIUS F. MINER, M. D.

Aneurism has for a long time received the attention of surgeons until it might seem that the most desirable plans of procedure would long since have been fully determined. Undoubtedly in most cases the best method of treatment is well established, but exceptions to general rules (if there can really be said to be any rules in surgery,) are so common as to justify a recital of successful methods, whether falling under established usages or not.

Wm. Kraengel, aged 27, sent for me in the early morning of August 25, with instructions that I come prepared to amputate his thigh, as he had pain too great for longer endurance. On arrival I found a resolute young man, suffering unbearable pain in the popliteal space, extending up to the thigh and through the hip joint. He had been recently a patient at Buffalo General Hospital, where he had been operated upon for popliteal aneurism. The external femoral artery had been ligated with temporary relief of pain, relief of pulsation, but not removal or diminution of size of tumor. Absence of pulsation, and of the bruit of aneurism made it impossible to determine whether suppurative action in the sac was the cause of pain, tenderness and tumor, or what seemed probable, that the aneurism was supplied at least from the anastomosing branches of the femoral, that when the external femoral was closed, the deep femoral enlarged to meet the requirements of the circulation and that thus the operation of ligaturing the external femoral, though temporarily affording some relief, did not permanently relieve the pressure upon the nerves of the part or diminish the size of the aneurismal tumor. It was decided to open down upon the tumor, determine as far as possible its character, and to act according to indications. An incision nine or ten inches in length fully exposed the tumor and showed its nature; the sac was of dark venous color, did not seem to pulsate when grasped by the hand, but in trying to raise it from its bed and separate it from the surrounding tissues, the fingers passed suddenly into the back part of the sac, which was softened to such an extent as to offer little resistance. The aneurismal coagulum was firmer than the walls of the sac; this was turned out of its bed and the fingers passed up to the opening of the vessels in the sac, thus controlling hemorrhage until a ligature could be passed around the vessels supplying the tumor. Hemorrhage, though at first profuse, was soon controlled, the sac emptied of coagulum, vessels ligated a few inches above the sac and one vessel from return circulation below the sac, the parts approximated by sutures and not very tightly applied bandage, and warm water dressings applied. In this operation I was assisted by Drs. Diehl, Dambach, Brush, W. W. Miner, and my private pupils Parker and Hopkins, and others. The result thus far is entirely satisfactory; pain gradually subsided from the time of operation, and no unpleasant effects have since been observed. The suppuration in the wound has been moderate in amount, and the secretions healthy in character. The ligatures were removed three weeks after the operation, and the parts completely closed except where the ligature rested. The popliteal nerve was found at the time of the operation, closely and inseparably adherent to the walls of the sac and some pain may reasonably be supposed to attend such condition of the nerve sheath.

It is no part of my purpose to discuss the various operations for aneurism. That opening down upon aneurismal tumors in the popliteal space, and ligating the vessels which supply them, rather than cutting off the circulation by ligation of common femoral, has some advantages I have no doubt; it may also be open to objections, which subject has been thoroughly discussed by George W. Norris, M. D., in his work entitled Contributions to Practical Surgery, in chapter upon mortality following ligature of arteries. This work contains the best resumé of the subject I have yet seen. I have for many years bestowed considerable thought upon the subject, attention being called to it by my first operation, now about twenty years since, for aneurism. This case was one of traumatic aneurism, at the bend of the elbow, about where the brachial artery divides into the radial and ulnar. Without at the time knowing very much what I was about to do, assisted by my former colleagues, Prof. Eastman and Dr. J. R. Lothrop, I opened down upon the tumor applied ligature to the vessels which supplied it, divided the vessels below ligature, and raising it from its bed, cut it out as fatty or cystic tumors would be removed. Return circulation afforded profuse hemorrhage, which was controlled by ligature to everything requiring it. Thus the diseased mass was removed, the wound closed, followed by complete recovery, without an unpleasant symptom. The complete success of the procedure though adopted in my inexperience, and I may say ignorance in such matters, has never failed to impress me with the conviction that aneurismal tumors at the bend of the elbow or in the popliteal space, may sometimes be more safely and certainly cured by extirpation than otherwise, and though I do not ask or advise others to follow my example, still I am willing to speak favorably of a procedure which has served me well when adopted, and which seems to me feasible and philosophical, and to have advantages worthy of consideration.

